# Ileostoma as a promising tool in pathophysiological study and clinical intervention: a review from bench to beside

**DOI:** 10.3389/fimmu.2026.1655512

**Published:** 2026-03-06

**Authors:** Yuchen Li, Ying Wang, Xiaolong Li, Lian Duan, Zemin Tian, Yuan Qiu

**Affiliations:** 1 Chinese People's Liberation Army (PLA) Medical School, Beijing, China; 2 Department of Neurosurgery, Chinese PLA General Hospital, Beijing, China; 3 Department of General Surgery, Xinqiao Hospital, Army Medical University, Chongqing, China; 4 Department of General Surgery, Chongqing General Hospital, Chongqing University, Chongqing, China

**Keywords:** early closure, ileostomy, immunotherapy, pathophysiology, postoperative complications, stimulation

## Abstract

Ileostomy is extensively utilized as a preventive method to avert anastomotic leakage and other severe complications in colorectal surgery, serving as a valuable model for both study and clinical applications. The ileostoma, as a window, allows for the observation of a range of pathophysiological changes of complications in the mucosal layer of the defunctioned ileum, as well as in the defunctioned colon. It is precisely the changes in the intestinal microecology caused by ileostomy that may affect the efficacy of anti-tumor immunotherapy. Various interventions exist to flush the defunctioned intestine via the window, aiming to mitigate both short-term and long-term complications. These interventions include the use of normal saline, short-chain fatty acids, autologous intestinal fluid, and probiotics. Furthermore, there is a growing perspective advocating for the early closure (EC) of the ileostomy as a potentially superior management strategy. Moving forward, continued exploration of the window promises to yield further benefits for patients.

## Highlights

- Ileostoma represents not only the anatomical window but also the temporal window.
- This review systematically described the pathophysiological changes of the defunctioned intestine and the complications that may result from these changes after ileostomy.
- There are many ways to intervene in clinical outcomes through ileostoma.
- Ileostomy may affect the efficacy of anti-tumor immunotherapy.

## 1 Introduction

Currently, to prevent pelvic sepsis due to anastomotic leakage in patients with colorectal anastomosis, a protective loop ileostomy is temporarily established until sufficient healing occurs. Throughout the interval from the ileostomy to the ileostomy closure, the intestinal contents are expelled through the stoma rather than the anus. Typically, this ileostoma remains in place for 2 or more months, until ileostomy closure is performed to facilitate the reestablishment of digestive tract continuity and the restoration of physiological fecal stream direction (1). According to global statistics, there is a high ileostomy adoption rate, even a trend of abuse. For example, in China, as a high-incidence country for low rectal cancer, the rate of ileostomy has surged to nearly 70–80%, a trend similarly observed in the USA and the UK (2–4).

However, after ileostomy, significant effects on the local intestine and whole body have been observed. In the ileum, the prevalence of early high-output ileostomy (defined as >1000–2000 ml/day) reaches approximately 16%, while symptomatic diversion colitis (DC) approaches 33% (5, 6). Furthermore, the closure of an ileostomy does not guarantee an end to associated challenges. The incidence of complications following ileostomy closure ranges from 17.3% to 21.5%, among which the most frequent complications are postoperative ileus (POI) (4.9–28.7%) and incisional surgical site infection (SSI) (3.1–40%) (7–15). Other complications include low anterior resection syndrome (LARS) (41–90%) and recurrent ileotic anastomotic leakage (3.3–13%) (16–21). In addition, despite the lower incidence of ileostomic and rectal stenoses, aggressive intervention is still required to prevent long-term complications. The occurrence of these adverse reactions greatly increases the physical, psychological, and social burden.

In addition, those achieved through immune checkpoint inhibitors (ICIs) are often used in anti-tumor immunotherapy, which is achieved by enhancing the immune activity of T-lymphocytes or suppressing the inhibition of the immune system. Clinically, monoclonal antibodies targeting programmed cell death protein 1 (PD-1), programmed death ligand 1 (PD-L1), and cytotoxic T lymphocyte-associated antigen 4 (CTLA-4) are generally used alone or in combination to achieve the purpose of anti-tumor. Among the immune-related adverse events (irAE) thus triggered, the incidence of lower gastrointestinal tract is the highest, often presenting as diarrhea or even colitis. For patients with an ileostomy, these immune-related adverse events are either absent or, at the very least, present with mild symptoms (22). However, conversely, whether the changes in intestinal immunity, including the microbiota and metabolites, caused by ileostomy will affect the efficacy of immunotherapy still needs to be explored.

Although the risk factors of these complications have been widely discussed, their fundamental mechanisms and management are not fully understood. Fortunately, since the patient had a certain period before ileostomy closure, the ileostoma can be considered an ideal window for us to study the inside of the intestine and can be used to conduct self-comparisons before and after ileostomy closure. Moreover, postoperative complications may be related to the pathology of the defunctioned intestine and associated with the cumulative effect of defunctioning over time (23–26). In recent years, some ileostoma-related changes have been gradually revealed, focusing on the interval (23, 25, 27–29). Meanwhile, attempts have also been made to study whether stimulating the defunctioned intestine via the ileostoma is beneficial for reducing postoperative complications and facilitating rapid functional recovery (30, 31). We further focused on the effects of changes in the defunctioned ileum and colon and the positive effects of interventions regarding ileostomy closure on the improvement of complications.

## 2 Pathophysiology after ileostomy

It is conceivable that changes in the histological structure of the intestine are often accompanied by functional impairments, resulting in different clinical manifestations. Here, we discuss the three critical aspects: the mucosal layer, neuromuscular layer, and defunctioned colon considering their anatomical position within the intestine. Through this window, we aim to elucidate the relationship between pathological changes and complications after ileostomy.

### 2.1 Mucosal layer

**Figure 1:**
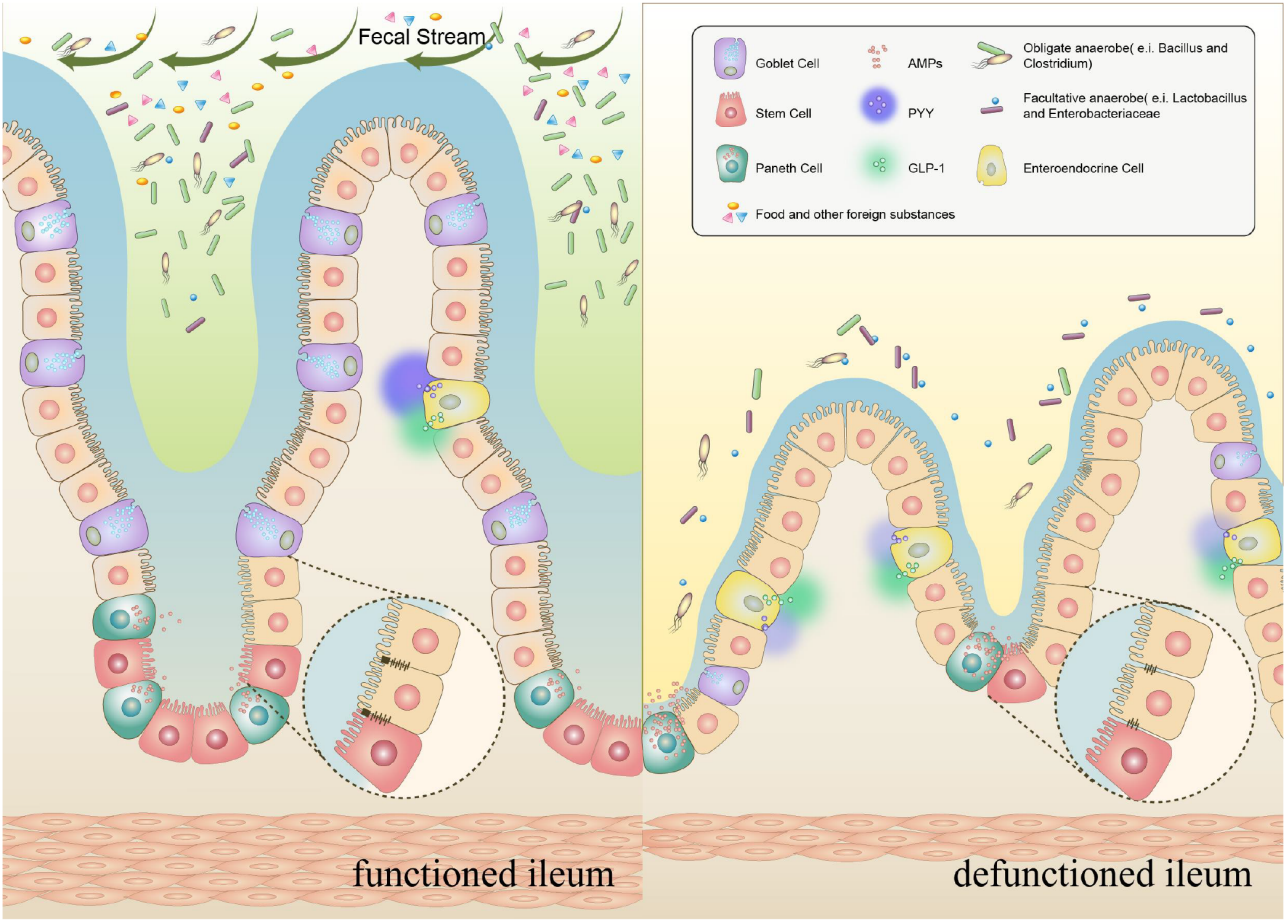
Pattern diagram of microscopic changes of intestinal villi at both ends of ileostomy.

**Figure 2:**
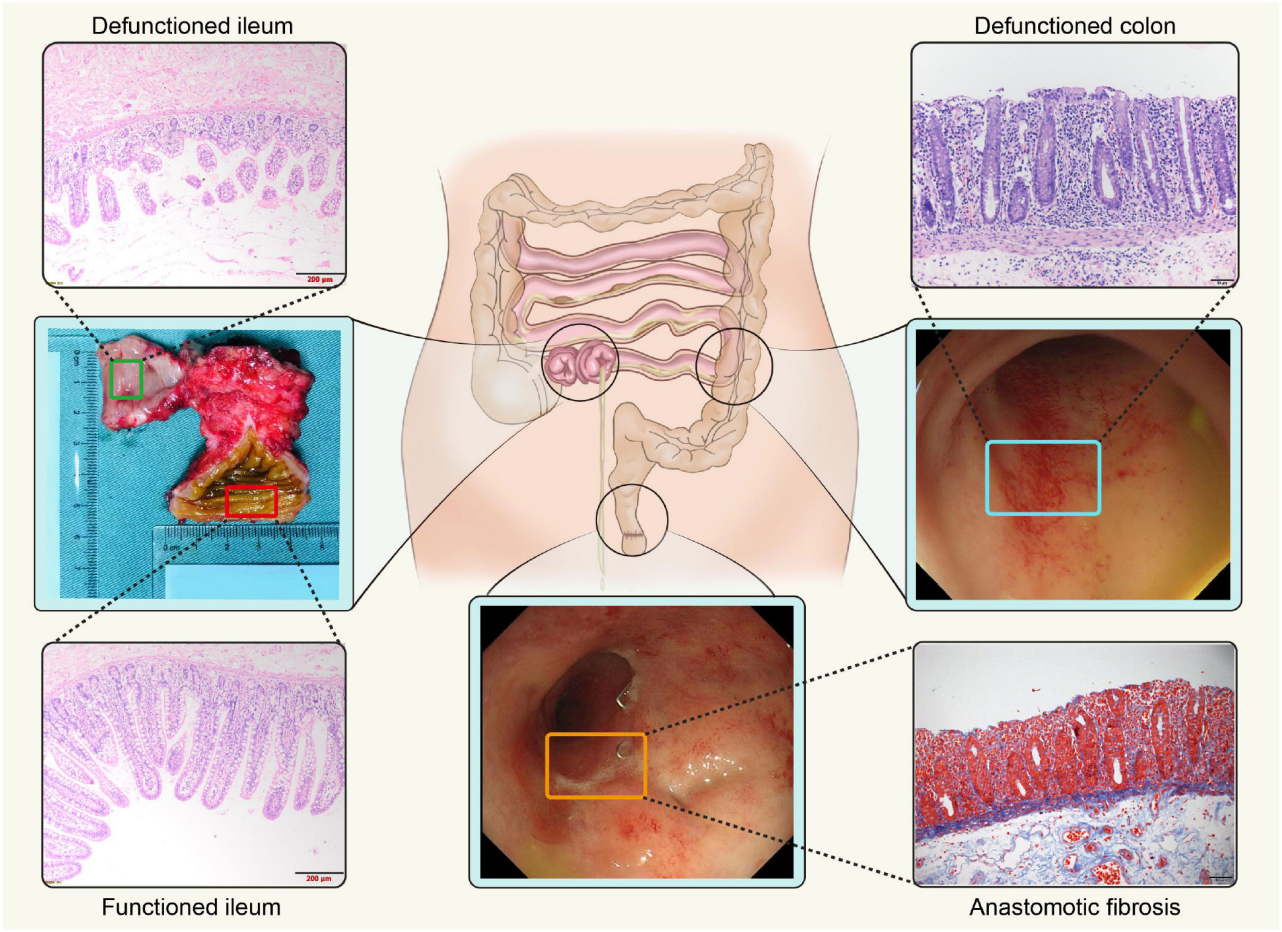
The endoscopic manifestations and histological changes after ileostomy in the study.

The efficiency of diverting the fecal stream in a well-structured loop ileostoma is nearly 100%. In this case, due to the lack of stimulation in the intestine, a series of changes, mainly atrophy, occurred in the mucosa (**Figure 1**). Over time, after long-term fecal diversion, the annular folds were apparently reduced and the ileal mucosa became smooth. In addition, we found that the mucus covering the mucosal surface was significantly reduced (24) (**Figure 2**). Likewise, the intestinal villus-crypt structures of the defunctioned ileum exhibit extensive atrophy, particularly evident in the height of the villi (23). In line with this, a study reported that the crypt depth of the defunctioned ileum was shallower than that of the normally functioned ileum (32). Within the spectrum of epithelial cells, goblet cells are important guarantors of intestinal lubrication. However, alterations in goblet cells in the defunctioned ileum are often significant. In addition, the single-cell sequencing study demonstrated a significant decrease in goblet cells in the defunctioned ileum, which was confirmed in other studies (33, 34). Beyond mere cell count, our investigations also unveiled a decrease in intracellular mucin granules and a diminished goblet cell volume (24, 34). Furthermore, our research indicates that mechanical stimulation enhances the expression of the key transcription factor SPDEF. This factor is instrumental in the differentiation and maturation of goblet cells, signaling through the TRPA1-ERK pathway. Such findings underscore the essential role of mechanical stimulation from the fecal stream in facilitating the terminal differentiation of goblet cells (34, 35). In contrast, Wieck et al. found no notable difference in the quantities of goblet cells or intestinal endocrine cells between functioned and defunctioned ileum (32). This may be associated with the fact that the aforementioned research adopted infant samples in the study and intestinal cells of infants may metabolize and multiply rapidly compared to adults. Furthermore, stem cells are located at the base of the intestinal crypt, and their continuous proliferation and differentiation ensure rapid renewal of the intestinal epithelium. Importantly, while the biomass of stem cells diminishes in the defunctioned ileum, their functionality remains largely unchanged. This resilience could be attributed to the inherent plasticity of stem cells that remain stored and dormant within the crypts (32, 36). In short, these studies proved that the fecal diversion only temporarily affects the proliferation of intestinal epithelial cells, and once feces is present again, the stem cells can be awakened and restored to function.

Similarly, the microbiota present in the defunctioned ileum has also undergone significant changes, which can be related to anti-tumor immunotherapy and complications. And the gut microbiome is divided into fecal flora and mucosal-associated bacteria (37). Recent evidence suggests that the abundance and diversity of defunctioned ileal microflora are significantly reduced. In particular, Beamish et al. demonstrated a 62.4% reduction in the mucosal-associated bacterial load alone, not including the total loss of fecal flora after loop ileostomy (25). Overall, the resulting imbalance of the intestinal flora may affect the efficacy of immunotherapy and increase the risk of drug resistance, studies have shown that due to the differences in the richness and species of the microbiota, there may be certain differences in the effects of immunotherapy (38, 39). Further studies have shown that the number of obligate anaerobes, such as Bacillus and Clostridium, decreased, whereas the number of facultative anaerobes of Lactobacillus and Enterobacteriaceae increased (28). The relatively increased Lactobacillus may synergize with Clostridium to produce indole-3-propionic acid (IPA). The product IPA enhances the immune level by increasing the acetylation of H3K27 in the super-enhancer region of Tcf7, regulating the stem cell program of CD8+T cells and promoting the production of CD8+T cells (Tpex) with exhausted progenitor cells. However, the impact on immunotherapy after ileostomy without adding special intervention is not clear because of decreased number of Clostridium (40). Disturbances in intestinal flora (especially the overall decrease in quantity) can also be an important factor in the development of complications (41). However, from another perspective, for example, the increased genus Veillonella which can be detected in the ileostomy is reported regarding non-small cell lung cancer (NSCLC) patients who are undergoing immunotherapy, especially with ICIs, seems to have some relation to the extension of survival time, and the same is true for colorectal cancer patients with ileostomy who are undergoing immunotherapy whether it is the same or not suggesting that the effect of immunotherapy may have been enhanced is worth discussing (39, 41). Overall, the changes in the microbiota after ileostomy may tend to have a net positive effect on the application of immunotherapy, which is consistent with our experience, but requires specific verification.

Typically, at the end of the ileum, there exists a 20–30 cm segment that is entirely defunctioned, leading to various challenges for patients with ileostomy, including disturbances in water, electrolyte, and metabolic imbalances. Beyond its local absorption functions, this terminal ileum region, abundant with endocrine cells, exerts systemic hormonal regulation, exemplified by peptide YY (PYY). Research indicates that PYY is predominantly situated within 30 cm of the ileocecal valve, precisely the area impacted by ileostomy (42). As a rule, PYY functions through a mechanism known as “ileal braking”. When unabsorbed nutrients reach the distal ileum, PYY secretion is triggered. This, in turn, influences the upper digestive tract by inhibiting gastric emptying, diminishing small intestine peristalsis, and reducing pancreatic secretions, thereby extending the transit time of chyme (43). The net effect is to optimize functioned intestinal absorption, including water and electrolytes, to reduce the occurrence of diarrhea (43). Therefore, a decrease in PYY may lead to rapid transport and reduced absorption of fluids, which is probably reflected in a high-output stoma and increased hunger observed during the initial period after ileostomy. However, there is a compensatory increase in PYY concentration in the normally functioned ileum at the upper end of the ileostoma, eventually stabilizing over time (42). Clinically, as time progresses, excretion tends to stabilize, with the effluent becoming thicker and more stool-like. These findings offer valuable insights into potential PYY-based therapies, such as its potential application in treating short bowel syndrome. However, significant challenges persist in translating these findings into clinical interventions. Furthermore, researchers have explored the ileostoma as artificial window to investigate glucose and intestinal hormone levels. Among various hormones, glucagon-like peptide-1 (GLP-1), primarily produced by intestinal L cells, has the capability to prolong gastric emptying time and reduce blood sugar concentration (44). Recently, Zhang et al. ingeniously utilized an ileostoma to infuse glucose solution into the defunctioned ileum. Their findings revealed that even a relatively short 30 cm exposure of the defunctioned ileum to glucose, administered over 60 minutes at a rate of 2 kcal/min, could significantly elevate plasma GLP-1 levels (45). This team also found that GLP-1 is regulated in a concentration-dependent manner and that the concentration level of GLP-1 is positively correlated with the concentration of actively absorbed glucose and is elevated by approximately twice as much as in non-flushed conditions. Why would the GLP-1 levels increase in this case? Several preclinical studies have linked glucose-induced GLP-1 secretion to Na+-coupled glucose transporter1 (SGLT1) (46). According to an *ex vivo* human ileal tissue study, the functional SGLT1 pathway is closely related, where the role of electrogenic sodium-dependent glucose uptake by SGLT1 is critical in causing membrane depolarization and subsequent glucose-induced GLP-1 secretion in human L cells (47). Other types of hormones are also being proposed for their potential to regulate the quality of life in patients with ileostomy (45). Future exploration of ileostoma-related hormones should concentrate on adding more types and combining them in various ways to alleviate complications. For example, a small amount of PYY injection and glucose solution stimulation in the defunctioned ileum could be combined, which may improve the symptoms of high output, eating frequency, and hunger.

It is also worth noting that considerable research efforts have been dedicated to understanding the impact of ileostomy on the intestinal barrier. Interestingly, most patients with ileostomy do not show significant enterogenic infection symptoms, suggesting relative stability in the intestinal barrier. According to Ralls, normal functioned ileal mucosa has significantly greater cross-epithelial resistance than a defunctioned ileum (48). In the defunctioned ileum, there is an increase in FITC-dextrin penetration, a notable decrease in the intensity of tight junction proteins ZO-1, Occludin, Claudin-4, and adhesion junction protein E-cadherin, along with impaired tight junction structure formation (48). Similar findings have been reported in animal models (49). Although the mechanism by which fecal diversion leads to the loss of epithelial barrier function (EBF) remains unclear, intraluminal factors are likely to be involved. It is believed that the defunctioned ileal mucosa only receives nutrients from the blood, losing nutrients from the chyme of the functioned ileum; therefore, the two ends absorbing distinct nutrients in different ways will alert the physical and chemical environment of the mucosa and the metabolism of epithelial cells. As described, from this point, fecal stream plays an integral role in maintaining the intestinal barrier. Pattern recognition receptor signaling is one of the main pathways through which the intestinal mucosa senses changes in the lumen (50). An emerging theory suggests that changes in the microbiota of the defunctioned ileum, including an increase in many gram-negative bacteria, may be the extrinsic manifestation of increased expression of pro-inflammatory cytokines in the mucosa and activation of TLR signaling, which leads to inflammation and barrier destruction (28, 48, 51). However, the specific factors maintaining the relative stability of the intestinal barrier in patients with ileostomy, especially given the infrequent occurrence of enterogenic infections linked to bacterial disruptions, remain somewhat elusive. This suggests the potential existence of alternative compensatory mechanisms. Here, the enhancement of the immune barrier, if not entirely, may be another protective mechanism. Our previous study demonstrated a significant increase in antimicrobial peptides, such as DEFA5 and DEFA6, secreted by Paneth cells in the defunctioned ileum after loop ileostomy, indicating enhanced intrinsic immunity against microflora dysbiosis in the defunctioned ileum (24, 52). Therefore, together with the two aforementioned findings, another interpretation could be that defective mechanical barriers increase the chance of content contact with epithelial cells, thereby altering microorganism-epithelial signaling and promoting an increase in immune- and defense-related biological processes in the diverted defunctioned ileum.

### 2.2 Neuromuscular layer

The neuromuscular layer is often a layer or area that is easily overlooked in intestinal studies but is greatly affected by ileostomy. In addition, some patients develop POI after ileostomy closure. One reason for this may be the lack of intestinal motivation, which mostly results from neuromuscular dysfunction. It is also associated with LARS after ileostomy closure (53). A multitude of studies have shown that long-term lack of lumen content and physical-mechanical stimulation may result in changes in the defunctioned intestinal basal layer, which is mainly reflected in the significant reduction in the strength of circular muscle (CM) contraction and smooth muscle area of the defunctioned intestine (23) (**Figure 1**). According to an animal study, in the defunctioned ileum, the number of neurons expressing vasoactive intestinal peptide, neuropeptide Y, or pituitary adenylate cyclase-activating peptide was gradually reduced; however, neurons represented by the intermuscular ganglia expressing nitric oxide synthase increased, which may be the basis for the fact that mechanical stimulation can alleviate defunctioned intestinal atrophy (54, 55). Overall, these results suggest that the weakened intestinal muscle layer, different release and expression of neurotransmitters in the defunctioned ileum nervous system, and decreased neural activity may better explain the diminished peristalsis, high relaxation responses, and frequent defecation.

### 2.3 Defunctioned colon

The effect of fecal diversion on the colon is also significant, with nearly 100% of patients often presenting with abdominal pain, mucus, hemorrhagic discharge, and posterior rigor. The endoscopic manifestations of DC are erythema, fragility, edema, nodules, avers ulcer, exudate, obvious bleeding, etc., which are similar to active idiopathic ulcerative and granulomatous colitis (56) (**Figure 2**). Histological changes mainly manifest as lymphoid follicular hyperplasia (57, 58). Interestingly, white blood cell count, neutrophil ratio, and C-reactive protein (CRP) levels in the mild DC group were normal, and only the CRP level in the severe DC group was elevated, suggesting that there may be an immune mechanism different from the general inflammatory response or that it may indicate an inflammatory response unrelated to infection (59). For severe DC, different studies have different evaluation methods, and no unified approach has been formed yet. Recently, there has been a report that the severity of DC is related to clinical symptoms and can be evaluated, which is different from previous reports (60). Therefore, severe colitis can be evaluated through multiple methods, including comprehensive histopathology (abnormal mucosal structure, deep tissue involvement), endoscopy (assessing mucosal damage through observation and scoring systems), clinical and laboratory tests. Additionally, for special populations, standards need to be adjusted to guide personalized treatment (61). Colon inflammation induced by cancer immunotherapy has similar pathological features to DC, but ileostomy ensures that patients do not have obvious symptoms (22, 62).

It is widely acknowledged that disturbances in the colonic microbiome significantly correlated with the severity of DC (63). Some researchers believe that the main causes of DC are the increase in aerobes in the defunctioned colon after fecal diversion, lack of short-chain fatty acids, and colonic immune disorders (5, 64). Another noteworthy point is that in a comparative study with healthy subjects, the defunctioned colon demonstrated a decrease in anaerobes, especially lactic acid bacteria and bifidobacteria (28, 63). Specifically, research on patients with colostomy indicates a significant reduction in lactic acid bacteria within the defunctioned colon compared to the microflora of the functioned colon (64). This transition may be due to the entry of oxygen into the normally anaerobic intestinal cavity following ileostomy surgery (28, 65). While shifts in bacteria like Staphylococci, Enterococci, Klebsiella, Pseudomonas, etc may not be as pronounced, they remain noteworthy (28). In addition, some researchers believe that ischemia may be another mechanism underlying DC formation (5). However, the further reason is closely related to the lack of SCFAs, including butyric acid, which can be produced by normal luminal bacteria, based on evidence that SCFA relaxes vascular smooth muscle and deficiencies may induce increased tone in the pelvic arteries, leading to relative ischemia of the colorectal mucosa and intestinal wall (66). SCFAs are the primary end products of the fermentation of non-digestible carbohydrates by the microbiota, among which butyrate is the principal oxidative substrate for colonocytes, and patients with DC may improve following topical treatment with SCFA, especially butyrate (67, 68). Evidently, the root explanation surely lies in changes to the luminal flora consequent to fecal stream interruption. Furthermore, a retrospective study involving 40 patients verified the correlation between the microbiota type and DC severity, utilizing 16s-rDNA sequencing technology to detect intestinal microorganisms; the edema and DC hemorrhage were compared based on endoscopic evaluations, which showed that intestinal flora with mild DC primarily consisted of Bifidobacteriales and Prevotella, whereas flora with severe DC consisted of Providencia and Dorea (60). Based on this theory, there is potential to alleviate the severity of DC by altering the microecological environment within the intestine or by supplementing the missing microorganisms and metabolites owing to fecal diversion. In contrast to DC, colitis caused by ICI also shows significant changes in diverse microbial flora, and different flora, in turn, can have a certain impact on the treatment of ICI (69, 70).

Apart from DC, approximately 80% ~ 90% of patients exhibit some symptoms of LARS after ileostomy closure, including increased frequency of bowel movements, incomplete bowel movements, difficulty in defecation, and fecal incontinence (71, 72). LARS can manifest as temporary (6–12 months) intestinal dysfunction, become long-term (12–18 months), or even evolve into permanent symptoms (73, 74). Furthermore, ileostomy is strongly correlated with LARS, doubling the risk compared to those without (75–78). In addition, rectal stenosis has increasingly been a challenge to the quality of life of patients after ileostomy, especially after the reconstruction of intestinal continuity. However, ileostomy as a preventive measure against anastomotic leakage is an independent risk factor for rectal stenosis (**Figure 2**). The natural process of DC includes persistent inflammation, which may facilitate the development of stenosis and the loss of colon function. Likewise, rectal stenosis is also more likely to occur after low anterior resection, with an incidence of 2–30%, mostly occurring within 6 months after surgery, and can also develop into a long-term complication (79–83). Quintessentially, the main clinical manifestations are poor bowel movements (i.e., constipation, colic, abdominal distension, and fuzzy indigestion) and blood in the stool. Digital rectal examination can reveal that the intestinal wall is narrow and hardened, mainly occurring above the anastomotic port, which may be tightly related to factors limiting movement, including intestinal muscle fibrosis, pelvic nerve damage, oxidative stress, etc. (84). Emerging studies suggest that late closure (LC) increases the risk of stenosis, and EC and functional neuromuscular recovery in the rectal region after ileostomy closure have been advocated. In general, dilation measures to destroy the proliferated fibrous tissue and antifibrotic drugs are recommended (85, 86). In the future, combination therapy of blocking the oxidative stress pathways related to stenosis, early and late use of antifibrin drugs, and expansion is worth exploring.

## 3 Interventions during the perioperative period of ileostomy closure

As described above, ileostomy causes fecal diversion and most of the stimulation of the distal intestine is lost. It is undeniable that most patients with ileostomy will receive significant benefits in improving their symptoms and prognosis if the defunctioned intestine is stimulated frequently and appropriately. Therefore, two essential aspects of intervention have been proposed: stimulation before ileostomy closure, and EC. This section provides a brief review of the corresponding impact of different preoperative stimulations and ileostomy closure time points on postoperative complications to contribute to clinical research and guidance.

### 3.1 Preoperative stimulation

Several studies have explored the stimulation of the defunctioned intestine before ileostomy closure, employing diverse protocols that range from using autologous fecal to physiological serum-containing thickening agents, short-chain fatty acids, liquid diets, and sucrose (**Table 1**). The potential benefits of preoperative stimulation were first reported by Ekelund and Williams (23, 54). The concept of functionally preparing the intestine before ileostomy closure is appealing, as it may decrease the period of intestinal dysmotility and microbial dysbiosis, as well as lessen the atrophy of muscular layers in the defunctioned intestine. This aligns with the notion that a “prepared” gut lowers the risk of complications (87).

**Table 1:** Studies regarding stimulating the defunctioned intestine.

No	Author & year	Country	Study type	Participants & details (SG vs CG)	Substance & dose (SG/CG)	Route of administration	Frequency & flushing time	Follow up time	Observation items	Results or findings of SG	Reference
1	Abrisqueta et al. (2014)	Spain	RCT	35 vs 35 Ileostomy patients for rectal cancer	physiological saline 500ml + thickening agent 30g/nonstimulation	Through ileostoma	Once daily; 2 weeks	After ileostomy closure	OT, PFS, POI, LOS, NG, TOA	Earlier return to OT and PFS, lower incidence of POI, shorter LOS, NG not required	(88)
2	Miedema et al. (1998)	USA	prospective cohort study	6 vs 7 Ileostomy patients with proctocolectomy and IPAC anastomosis for chronic ulcerative colitis	Isotonic saline sucrose solution 100ml/nonstimulation	Through ileostoma	Twice daily; 6 weeks	With treatment going and after ileostomy closure	ABS, MOT, FOM, OT, PFS, LOS, DR	No significant improvement in water, electrolyte, vitamin B12 Abs or Mot, FOM, PFS, LOS; A trend to earlier liquid OT	(90)
3	Fernández López et al. (2019)	Spain	prospective cohort study	16 (SG) Ileostomy patients for rectal cancer	SCFA solution 300ml (propionate, butyrate, acetate)/NA	Through ileostoma	Once daily; 7 days	After ileostomy closure	NRA, AE, PFS, OT, LOS, POD	Less NRA, no AE, earlier return to OT and PFS, shorter LOS	(91)
4	Rodríguez-Padilla et al. (2021)	Spain	RCT	34 vs 35 Ileostomy patients for rectal cancer	physiological saline 250ml + Probiotics 4.5mg (Vivomixx^®^, lyophilized live bacteria)/physiological saline 250ml	Through ileostoma	Once every two days; 20 days	After ileostomy closure	POI, NG, OT, LOS, PFS	No significant statistical improvement in observation items	(97)
5	Marcelino et al. (2023)	Brazil	RCT	52 vs.52 Ileostomy patients for rectal cancer	saline solution 500 ml + probiotics 6g (Simbioflora^®^, fructooligosaccharide with probiotics)/nonstimulation	Through anus	Once daily; 15 days	Weekly with treatment going and 7–14 days, 1, 3, 6 months after ileostomy closure	PFS, NG, OT, Cost, LOS, GOC, LARS, SC, NV, OCDH, FI, ACP, NE, QOL	NA	(92)
6	Ocaña et al. (2022)	Spain	prospective cohort study	24 vs 34 Ileostomy patients for rectal cancer	fecal contents from the ileostomy bag 200ml/nonstimulation	Through ileostoma	NA; 15 days	After ileostomy closure	POI, PFS, OT, LOS, AL, SSI, IADA, EVI, SBP, PNE, IAB, CRP, BMI	Earlier return to liquid OT and PFS, lower incidence of POI	(31)
7	Duan et al. (2019)	China	prospective cohort study	26 vs 26 Ileostomy patients for Crohn’s disease	chyme collected from the functioned ileum/nonstimulation	Through ileostoma	NA; NA	After ileostomy closure	POI, POD, LOS, PFS, SSI, BMI, TOA	Lower incidence of POI and rate of POD, shorter LOS	(87)
8	Liu et al. (2021)	China	retrospective cohort study	30 vs 42 Ileostomy patients for rectal cancer	fresh succus entericus diluted by 500–1000 ml normal saline/nonstimulation	Through ileostoma	NA; 14–28 days	With treatment going and after ileostomy closure	POI, PFS, LOS, LARS, OPT, BL, POD, ALB	Earlier return to PFS, shorter LOS, lower LARS score in 1 week, no significant improvement in POI	(95)

SG, stimulation group; CG, control group; RCT, randomized controlled trial; SCFA, short chain fatty acids; NA, not applicable; OT, oral tolerance; PFS, passage of flatus or stool; POI, postoperative ileus; LOS, length of hospital stay; NG, nasogastric tube; ABS, absorption; MOT, motility; FOM, frequency of movement; NRA, need for rescue analgesia; AE, adverse effect; GOC, grade of colitis; LARS, low anterior resection syndrome; SC, stool consistency; AL, anastomotic leak; SSI, surgical site infection; POD, postoperative diarrhea; TOA, type of anastomosis; DR, death rate; NV, nausea and vomiting; OCDH, operative complications during hospitalization; FI, fecal incontinence; ACP, abdominal cramps/pain; NE, number of evacuations per day; QOL, quality of life; EVI, evisceration; SBP, small bowel perforation; PNE, pneumonia; IAB, intraabdominal bleeding; IADA, intraabdominal distant abscess; CRP, C-reactive-protein values; BMI, body mass index; OPT, operation time; BL, blood loss; ALB, albumin.

Among all these methods, flushing with saline may be a simple and prevalent choice. In 2014, promising outcomes were observed in a single-center randomized trial. In this study, stimulation with 500 ml of normal saline combined with 30 g thickener to individuals via the efferent ileostoma for two weeks preceding ileostomy closure led to a reduced incidence of POI and an effective reduction in the postoperative period of hospital stay and dietary intolerance (88). Subsequently, the benefits were proven in the first multicenter randomized controlled trial using the method described above (89). However, in some cases, daily administration of isotonic saline sucrose solution does not improve ileal function in patients who undergo ileal pouch-anal canal anastomosis (IPAC), which may be related to the insufficient length (approximately 25 cm) of the stimulated defunctioned ileum segment (90). Therefore, normal saline can be understood as a mechanical stimulation that can carry various substances, and the functional outcome requires a certain length of the intestine as the basis. In addition to simply infusing saline, researchers have studied the substance richness, including chyme, feces, and various probiotics. Perfusion of the defunctioned ileum with SCFA can lead to earlier recovery of bowel function by maintaining intestinal mucosal homeostasis (91). The use of probiotics in clinical trials is a bold and innovative approach. Currently, there is a practice of administering a rectal flush comprising 500 ml of saline solution mixed with 6 g of fructooligosaccharide and probiotics (Lactobacillus acidophilus, Lactobacillus rhamnosus, Lactobacillus para casei, Bifidobacterium lactis) (Simbioflora^®^) before the ileostomy closure. This approach aims to ascertain its potential benefits in reducing the incidence of adynamic ileus (92). A more physiologically appropriate or explicit approach is to flush the defunctioned intestine with a filtrate derived from the fluid/chyme proximal to the ileostoma or using autologous feces, given its intricate composition. Notably, for patients with Crohn’s disease who undergo ileostomy, research has indicated that introducing one’s own bowel fluid may exacerbate the severity of the condition (93). However, updated data have confirmed that some advantages have not been explored. A comparative study on ileostomy patients with Crohn’s disease indicated that chyme reinfusion (CR, collected from the upstream stoma every 2–4 h and filtered with a three-tier gauze) into the downstream intestine could effectively reduce the incidence of postoperative diarrhea and POI, as well as shorten the postoperative hospital stay after ileostomy (87). This might be associated with the fact that CR can improve nutritional status and prevent intestinal failure before ileostomy closure (94). Furthermore, research indicated a notable decrease in the incidence of POI, along with expedited restoration of bowel function (1 day vs. 3 days) and faster adaptations to liquids (1 day vs. 2 days). These positive outcomes were observed when patients self-administered a daily stimulation for 15 days before ileostomy closure, using 200 ml of fecal content from the ileostomy bag via efferent ileostoma (31). There have been reports of self-administered succus entericus reinfusion (95). Additionally, recent findings suggest that using a probiotic formula (Ecologic^®^825, including 9 probiotics) for managing ileostomy-related flora, can lead to a notable reduction in pathogenic bacteria, enhanced proliferation of probiotics, and modulation of the metabolic environment through dynamic microbial interactions (96). This experiment is also an example of using the window for intervention, and it is expected to develop more personalized probiotic formulas for different cases. However, some researchers remain skeptical of this approach, noting its time-intensive nature. For example, studies suggest that probiotic stimulation via the ileostoma may not effectively reduce the incidence of POI (97). Despite this, there was a simultaneous reduction in both macroscopic and microscopic colitis by flushing with probiotics, as well as a decrease in short-term symptoms after ileostomy closure. Employing a spectrum of methods to address the defunctioned intestine—ranging from simple mechanical stimulation to intricate formulations—appears to significantly enhance patients’ quality of life. Similarly, the method of fecal microflora transplantation (FMT) regulating the effect of immunotherapy also has broad clinical prospects (98, 99).

### 3.2 Selection of time points for ileostomy closure

In past clinical practices, ileostomy closure typically occurred around two or more months, termed as LC (100). However, during this period with patients living with a stoma, the incidence of complications related to ileostomy ranged from 2.9% to 81.1%, adversely affecting their quality of life (101). Presently, an increasing number of surgeons advocate for EC (**Table 2**). Many researchers support the idea that EC approximately represents intestinal reconstruction within 30 days after ileostomy. Moreover, from a certain point of view, restoring intestinal patency as soon as possible can be also regarded as an early continuous physiological stimulation to the defunctioned intestine. According to a cross-sectional study of patients and surgeons, approximately 50% of patients believed that ileostomy had a significant impact on their quality of life, 72.9% preferred EC, and 80% of surgeons were willing to participate in randomized controlled studies to determine whether EC is clinically advocated (102).

**Table 2:** Summary of studies included in the comparison between EC and LC.

No	Author & year	Country	Study type	Participants & details (EC vs. LC)	Interval mean (range) (EC)	Interval mean (range) (LC)	Follow up time	Observation items	Results or findings	Reference
1	Danielsen et al. (2017) Park et al. (2018)	Danish and Swedish	RCT	55 vs. 57 Ileostomy patients for rectal cancer	11days (8-13days)	148days (>12weeks)	Before and at closure, 3, 6, 12 months after ileostomy closure	AL, LOS, SSI, NOC, DOS, BLE, MOA, EA, PFS, OT, NV, CP, LI, P, ALE, PAN, REO, FAI, SBO, STE, HVO, SI, SU, PH, PRO, RET, QOL, AB	NOC of EC decreased during follow-up, no significant difference in QOL	(103, 105)
2	Lee et al. (2019)	Korea	prospective cohort study	24 (EC) Ileostomy patients for rectal cancer	13.1days (8-16days)	NA	After ileostomy closure	AL, SSI, LOS, MOA, OPT, AB, ILT, ORS	closed safely two weeks after ileostomy, no patient developed any complications except 1 case of SSI, and no delay in adjuvant chemotherapy	(106)
3	Bakx et al. (2003)	Netherlands	prospective cohort study	18 vs. 9 Most ileostomy patients for rectal cancer	11days (7-21days)	NA	29 weeks (5–225 weeks) after ileostomy closure	AL, SBO, SSI, GP, DRE, POR, LR, ICS, LOS, DOS, P	EC is feasible and the incidence of complications is low and the complications were mild	(107)
4	Menegaux et al. (2002)	France	prospective cohort study	10 vs. 19 (14 vs. 22, Jejunostomy added)All ileostomy patients were considered for early closure on postoperative day 10 if feasible	10days	84days (56-168days)	After ileostomy closure	DR, AB, LOS, PFS, OT, STE, REO	without severe complications and significantly reduced the length of hospital stay	(108)
5	Alves et al. (2008)	France	RCT	95 vs. 91 Ileostomy patients with LA	8days	60days	1, 2, 3, 6 and 12 months after the rectal resection	DR, REO, LOS, SBO, AL, PNE, SSI, UTI, LYM, DVT, PFS, QOL, STE, BLE, AB	EC is feasible with reduced LOS, POI and medical complications, but a higher wound complication rate	(109)
6	Baik et al. (2021)	Korea	retrospective cohort study	354 (LC) ileostomy patients for rectal cancer and other conditions	NA	130days (30-1089days)	After ileostomy closure	SSI, SBO, AL, PNE, PC, VD, ALB	LC is a risk factor for morbidity of ileostomy closure	(120)
7	Elsner et al. (2021)	Switzerland	RCT	37 vs. 34 Ileostomy patients for rectal cancer	15days (10-134days)	89days (76-128days)	6 weeks and 4 months after ileostomy, but stopped for safety concerns	QOL, AL, REO, ITOO, ADH, ICF, BL, OPT, ID, OT, PFS, LOS, POI, POD, HVO, SSI, PNE, UTI	ITOO, ADH, AL, and REO were significantly higher after EC, no difference in QOL	(111)
8	Vogel et al. (2023)	USA	RCT	10 vs. 12 Ileostomy patients with IPAA for ulcerative colitis	11days (8-12) days	59days (45-182days)	30 days after ileostomy closure, but stopped after interim analysis	REO, MOA, OPT, POI, AL, LOS, ICF, BAC, IPAAL	The complication rate of EC is high, some patients have serious complications, and the risk of readmission is high	(112)

EC, early closure; LC, late closure; RCT, randomized controlled trial; LA, low colorectal, coloanal or ileoanal anastomosis; IPAA, ileal pouch anal anastomosis; Interval, the period from ileostomy to ileostomy closure; NA, not applicable; AL, anastomotic leak; SSI, surgical site infection; LOS, length of hospital stay; NOC, number of complication; DOS, duration of surgery; BLE, bleeding; MOA, method of anastomosis; EA, epidural anaesthesia; PFS, passage of flatus or stool; OT, oral tolerance; NV, nausea and vomiting; CP, cardiopulmonary; LI, liver insufficiency; P, pain; ALE, allergy; PAN, pancreatitis; REO, reoperation; FAI, failed attempt of ileostomy closure; SBO, small bowel obstruction; STE, stenosis; HVO, high volume output; SI, skin irritation; SU, stomal ulcer; PH, parastomal hernia; PRO, prolaps; RET, retraction; QOL, quality of life; AB, abscess; OPT, operation time; ILT, Intraoperative leakage test; ORS, operation name of radical surgery; GP, gastroparesis; DRE, delayed recovery; POR, postoperative radiotherapy; LR, logistic reasons; ICS, intravenous-catheter sepsis; DR, death rate; PNE, pneumonia; PC, pseudomembranous colitis; VD, voiding difficulty; ALB, albumin; ITOO, intraoperative tendency of oozing; ADH, adhesion; ICF, ileostomy closure failure; BL, blood loss; ID, intestine diameter; POI, postoperative ileus; POD, postoperative diarrhea; LYM, lymphangitis; DVT, deep vein thrombosis; UTI, urinary tract infection; BAC, bacteremia; IPAAL, IPAA leak.

According to many studies, both the notable effect on the improvement of early intestinal complications and the safety of EC are issues that need to be addressed. Numerous studies have been conducted to support EC by providing a robust evidence base (**Table 2**). For example, a multicenter Randomized Controlled Trial analyzed 112 patients undergoing ileostomy closure at different intervals. The study revealed that EC within 8–13 days is not only safe but also results in a lower incidence of complications within 12 months post-index surgery compared to LC (>13 w), lending significant credibility to this approach (103). Similarly, a meta-analysis with firm evidence suggested that patients with EC have a lower incidence of POI, shorter overall operative time, and better quality of life (104). Other studies have similarly demonstrated the safety and benefit of EC in preventing reoperation, readmissions, and severe complications (105–109). Thus, the advantages and feasibility of EC are evident. However, in clinical practice, some issues are associated with EC. Several studies have shown that EC leads to an increase in early postoperative complications, which is the main cause of patient safety issues. For example, a meta-analysis highlighted a notably higher incidence of superficial incisional surgical site infections (SSI) among EC groups (EC: <30days) (104). Furthermore, EC occurs within 2 weeks, which further increases the chances of various intraoperative and postoperative complications (110). A study showed that the intraoperative tendency of oozing, adhesions, leakage of colonic anastomosis, leakage of colonic or ileal anastomosis, and reintervention were significantly higher in patients with cancer who underwent low anterior resection after EC (111). And it could result in severe complications in patients with ulcerative colitis who underwent ileal pouch-anal anastomosis (IPAA) after EC (112). Among these problems, the healing of the rectal anastomosis is probably the most important one that should be considered for EC (113). In fact, in terms of the patient’s postoperative quality of life, specifically high-power prospective randomized studies are required to definitively assess whether EC mitigates the development of LARS. A significant number of patients had to receive adjuvant chemotherapy after surgery, which may be the reason for their extended closure of the ileostoma. Therefore, whether fecal diversion from ileostomy affects the effect of adjuvant chemotherapy is a question worth exploring (114). Thus, EC may be more beneficial to the patient when it’s confirmed that they tolerate it within two weeks to a month, or even sooner. However, despite its safety being validated through clinical evaluation, EC has not been widely adopted in most cases.

Because the benefits of EC have been discussed, it is imperative to discuss the potential drawbacks of LC. Regardless of the short- or long-term perspectives, LC is less favorable for the recovery of intestinal function and is more prone to increase the incidence of related complications, especially severe ones. Among all postoperative complications, POI is likely to have the most significant impact and highest incidence, and is associated with increased length of hospital stay, risk of infection, and higher healthcare costs (115–117). A longer interval surgery length may be a risk factor for POI (118). If so, this would have a significant impact on our clinical protocol adjustment. However, LARS, which is generally responsible for poor quality of life after ileostomy closure, is also affected by the length of the interval. One study found that patients with an ileostoma > 6 months increased risk of LARS by 3.7-fold (119). In addition, an increased risk of LARS associated with prolonged ileostomy closure was supported by a meta-analysis (78). Similarly, another piece of evidence suggests that longer intervals may increase the incidence of early complications, including wound infection and small intestine ileus, after ileostomy closure with a certain possibility (120).

In summary, for temporary ileostomy, under the premise of ensuring the quality of life and the safety of closing the ileostomy, EC is easier to recover patient function, and the probability of complications tends to be lower. While some studies advocate for shorter ileostomy closure times, variability in data across studies prevents definitive conclusions about the benefits of EC and further exploration is still needed.

## 4 Conclusion remarks and future perspectives

In view of the current situation and the development of the colorectal surgery, ileostomy is a procedure that will not be replaced and is still very common in clinical practice. In this discourse, we introduce the concept of the ‘window’—a term that encapsulates not just an anatomical dimension but a temporal dimension. It transcends being merely a conventional surgical preventive or palliative measure against anastomotic leakage; rather, it beckons for deeper exploration to uncover underlying pathological mechanisms and refine clinical intervention protocols. The discovery of basic research, novel substances, and methods can further provide new inspiration for flushing the defunctioned intestine and optimizing its structure from both micro- and macro-perspectives. Moreover, the potential of this ‘window’ becomes even more compelling when leveraged to elucidate the intricate interplay between host and gut bacteria (121). Experiments involving the ingestion of probiotics or some specific drugs orally have harnessed the properties of this window for detection purposes (122, 123). Concurrently, forthcoming endeavors could involve introducing specific bacterial strains into the intestinal lumen via this ‘window’. Such interventions aim to discern the nuanced impact of particular microorganisms on defunctioned segments of the ileum or colon, thereby shedding light on the gut bacteria and their correlation with host. As the current focus in this regard mainly lies in the impact of intestinal flora (or rather, tumor microenvironment) on anti-tumor immunotherapy, the ileostomy, a good window for conducting experiments, should be given more attention and utilized (124–126). Although the targets and mechanisms of anti-tumor immunotherapy are gradually being revealed (40, 127), there is still no direct clinical trial evidence for the connection between ileostomy and immunotherapy, and extensive research still needs to be carried out through this ‘window’.
